# Economic Evaluation of Multisystemic Therapy for Young People at Risk for Continuing Criminal Activity in the UK

**DOI:** 10.1371/journal.pone.0061070

**Published:** 2013-04-22

**Authors:** Maria Cary, Stephen Butler, Geoffrey Baruch, Nicole Hickey, Sarah Byford

**Affiliations:** 1 King’s College, London, United Kingdom; 2 University College of London, London, United Kingdom; 3 Brandon Centre, London, United Kingdom; 4 Imperial College, London, United Kingdom; The University of Queensland, Australia

## Abstract

**Objective:**

To evaluate whether multisystemic therapy (MST) is more cost-effective than statutory interventions that are currently available for young offenders in England.

**Method:**

A cost-offset evaluation of MST based on data from a randomised controlled trial conducted in North London, England, comparing MST with usual services provided by two youth offending teams (YOT). Service costs were compared to cost savings in terms of rates of criminal re-offending.

**Results:**

108 adolescents, aged 11–17 years, were randomly allocated to MST+YOT (n = 56) or YOT alone (n = 52). Reductions in offending were evident in both groups, but were higher in the MST+YOT group. At 18-month follow-up, the MST+YOT group cost less in terms of criminal activity (£9,425 versus £11,715, p = 0.456). The MST+YOT group were significantly cheaper in terms of YOT services than the YOT group (£3,402 versus £4,619, p = 0.006), but more expensive including the cost of MST, although not significantly so (£5,687 versus £4,619, p = 0.195). The net benefit per young person for the 18-month follow-up was estimated to be £1,222 (95% CI −£5,838 to £8,283).

**Conclusions:**

The results reported in this study support the finding that MST+YOT has scope for cost-savings when compared to YOT alone. However, the limitations of the study in terms of method of economic evaluation, outcome measures used and data quality support the need for further research.

## Introduction

Treatment of young offenders has become a critical issue on the policy agenda, mainly because of the considerable social and economic costs incurred by this population. Crimes committed by young people are a substantial financial burden for the criminal justice system and for education, health and social services.[1]

Serious and repeated anti-social behaviour during adolescence can persist into adulthood, with conversion rates from childhood conduct disorder to adult antisocial personality disorder estimated to be between 40 and 70%.[2] Effective prevention strategies are therefore required.

Multisystemic therapy (MST) is an intensive home and community-based intervention that uses evidence-based interventions to intervene directly in the systems and processes related to the young person’s antisocial behaviour, for example, low parental monitoring, low school involvement and delinquent peer association.[3] Outcomes include reduced offending and antisocial behaviour, improved family, peer and school functioning and the prevention of the young person being placed out of home.[4], [5] MST was developed by the family services research centre at the medical university of South Carolina as an alternative to usual mental health services; these usual mental health services were found to be expensive and of low effectiveness.[6]

MST has been extensively evaluated in randomised clinical trials (RCT).[7] The majority of studies have been conducted by the developers in the USA and consistently demonstrate that MST is more effective than treatment as usual (TAU) in reducing youth re-offending, preventing out of home placement and improving individual and family functioning. However, a systematic review of eight studies was less positive. No significant difference between MST and TAU for restrictive out-of-home placements, arrests or convictions was found.[7] This review included a study conducted in Canada that failed to demonstrate the effectiveness of MST over TAU.[8] Until recently, little was known about the effectiveness of MST in the UK. The relevance of results from US studies for countries with different social, cultural, legal and mental health contexts cannot be assumed.

This paper reports the results of a cost-offset evaluation of MST based on a randomised controlled trial conducted in North London, England.[9] The trial evaluates the effectiveness of MST in reducing re-offending among young offenders compared with usual services provided by youth offending teams (YOT). The provision of MST, if effective, would require a shift in resources that could be used elsewhere in the health system. As a result, the economic consequences of this intervention need to be determined.

## Methods

### Participants and study design

Full details of the study methods are reported elsewhere.[9] In brief, the trial recruited consecutive referrals from two local youth offending services in North London between November 2003 to December 2009. Young people were included in the study if they met the following criteria: age between 13 and 17 years; living in the home of and being brought up by a parent or principal caretaker; and on a court referral order for treatment or a supervision order of at least 3 months’ duration, or, following imprisonment, on license in the community for at least 6 months. They were excluded if they met the following criteria: were a sex offender; presented only with substance misuse; were diagnosed with a psychotic illness; or posed a risk to trial personnel. They were also excluded if there was incompatible agency involvement (e.g., ongoing care proceedings). Consent forms were obtained before study entry from the next of kin, caretakers, or guardians on the behalf of the children participating in the study and adequate time to consider participation was given to the family.[9]

Participants were randomly allocated to either MST plus YOT support or YOT support only using a stochastic minimization programme (MINIM) balancing for type of offending (violent versus non-violent), ethnicity (white versus other) and gender.

Ethical approval was given by Camden & Islington Community Local Research Ethics Committee (05/Q0511/19). This trial was registered in ClinicalTrials.gov database with the registration number NCT01713088.

### Interventions

#### MST

MST is a family- and community-based intervention that establishes close contact with families to understand and deal with the factors that cause the young person’s antisocial behaviour. The intervention targets the individual’s adjustment, family relationships, school functioning and peer group affiliations. Therapists help caretakers develop skills to intervene and operate changes in important domains such as young person’s individual adjustment, their family relationships, school functioning, and peer group affiliations.[2]

The MST programme is licensed by MST-services, Inc (Charleston, SC). The team that delivered MST as part of this trial participated in MST Services’ quality assurance procedures.[4] This team comprised three therapists, with master’s level qualification in counselling psychology or social work, and a supervisor. Therapists had small caseloads, were available 24 hours a day, seven days a week for the families in treatment and visited them 3 times a week.[9]

#### YOT (usual services)

YOT intervention consisted of services currently available to young offenders in accordance with the Youth Justice Board National Standards.[9] These services included supporting the young person to re-engage with education, with substance misuse problems and anger management; training them in social problem-solving skills; and programs to decrease vehicle-crime, violent-offending and knife crime. The treatments were delivered by professional social workers, specialist therapists or probation officers.[9] The duration of both treatments was variable, but on average lasted 5 months.

#### Outcome measure

Outcomes were assessed at 6-monthly intervals: for the 6 months before randomization, for the 6 months covering the intervention period, and then every 6 months until the 30-month follow-up point. The primary outcome measures for the clinical trial were rates of offending behaviour. As this is also the primary concern of policy makers funding MST it was chosen as the primary outcome measure for the economic evaluation. Data on offending behaviour were obtained from the Young Offender Information System (YOIS) database, which records detailed offence information, court appearances, criminal orders, police custody records and arrest rates.[9] The data collected were categorised into violent and non-violent offending behaviour.

#### Resource use and costs

The results of the economic evaluation are reported from the perspective of the youth offending team and wider criminal justice system plus the cost of the MST intervention, (currently funded by the Department of Health). Data on MST contacts were collected from therapist records. Data on services provided by the YOTs were collected for the treatment period only (at first 6-month follow-up). These services included appointments with social workers, reparation workers, drug workers, connexions workers, parenting workers, group workers and psychologists. No data on YOT services were available for subsequent follow-up periods so the level of service use was assumed to remain the same as for the first 6-month follow-up period.

All unit costs, in UK pound sterling, were for the financial year of 2008–2009, the most recent financial year over which the trial data were collected. Unit costs were uprated to 2008/09 prices if necessary using the Retail Price Index.[10] The UK Treasury discount rate of 3.5% was used to discount both costs and outcomes. [11]

Intervention sessions were costed on the basis of the salary of the MST professionals involved. A cost per-hour was calculated which included all relevant employer costs (National Insurance and superannuation contributions), appropriate overheads (capital, administrative and managerial) and the cost of the supervisors time.[12], [13] Intervention sessions lasted approximately 60 minutes, and indirect time (for example, supervision, training, preparation and writing up notes) was included using information provided by the trial therapists on the ratio of direct face-to-face contact to all other activities. Travel costs to home visits were also included.

National unit costs were applied to YOT services and criminal activity.[14] The offending data collected included the number of records of violent and non-violent offending behaviour, but did not detail the exact nature of the offences committed. Unit costs for criminal activity are provided by type of offence.[14] These values include costs in anticipation of the crime, costs as a consequence of the crime and costs of the response to the crime. Violent offences were costed on the basis of the cost of an episode of violence against the person (£12,267)[15]. The cost of non-violent offences was estimated in two steps. First we computed the weighted average of non-violent offences for the age range of the young persons included in the study, using the 2006 cross-sectional sample from the Offending, Crime and Justice Survey.[16] This sample includes over 5,000 individuals aged 10–25, is weighted to be UK representative and details mean number of offence per person by age and for different types of offences.[14] Second, the weighted average for each type of non-violent offence was multiplied by the relevant unit cost.[17] The mean of these weighted average costs was applied to each instance of a non-violent offence (£1,100).

### Analysis

Economic analysis was carried out on an intention-to-treat basis. No generic measures of quality of life were included in the study so it was not possible to undertake a cost-utility analysis to answer the broader research question of whether the additional cost of MST can be justified in comparison to other possible uses for the funding. Given a strong policy preference for a focus on reductions in criminal behaviour and the availability of relevant data, a cost-offset framework was used to assess the economic benefit of MST. This involves comparing the additional cost involved in the provision of the new intervention (MST) with savings that may be generated by the new intervention (in this case in terms of criminal activity), in comparison to the alternative (YOT).

Primary analyses compared the incremental cost of services (MST plus YOT services) with incremental savings in relation to criminal activity over the 18-month follow-up for the sample of young people with complete economic data. A substantial amount of data was missing at the final 30-month follow-up point, as not all patients had completed their assessments, therefore this time point was only explored in a secondary analysis. Missing data was explored in one-way sensitivity analysis using a number of alternative methods, including last value carried forward, imputation using the mean of responses in the relevant group and imputation using the median.

Analyses compared the mean costs in the two groups using standard t-tests with ordinary least squares regression used for adjusted analyses and the validity of results confirmed using non-parametric bootstrapping[18] to enable inferences to be made about the arithmetic mean.[19] The prognostic variables used for baseline adjustment were treatment, age, gender, mean cost of all offences committed at baseline (6 months prior randomization), ethnicity, parent and peer attachment using the Inventory of Parent and Peer Attachment (IPPA) questionnaire[19] and socio-economic status (SES). The IPPA questionnaire is a self reported scale that measures adolescents’ perception of their attachment to peers and parents. Higher scores indicate higher quality attachment to parents and peers.[9] For SES a scale integrating information on parent education (six categories from none to higher degree) and occupation (six categories from without income to professional employment) was used. This scale ranges from 0–6, with a higher score being associated with higher SES.[9]

## Results

### Participants

108 adolescents, aged 11–17 years, were randomly allocated to MST+YOT (n = 56) or YOT (n = 52). Full economic data were available for 91 individuals (84%), 46 in the MST+YOT group and 45 in the YOT group.

A comparison of baseline characteristics (treatment group, age, gender, ethnicity, SES, cost of baseline offences and IPPA score) between those included in the economic evaluation and those for whom data was missing revealed no statistically significant differences. In addition, there were no significant differences in baseline characteristics between the two randomised groups. Table 1 shows demographic and clinical details of the sample included in the economic evaluation. The majority of the participants were male (82%), white (60%) and from a low socio-economic background (mean SES 2.3). The mean cost of offences recorded in the 6 months prior to randomisation was higher for the MST group, due to a higher number of violent offences reported. The full trial profile and description of the participants has been published elsewhere.[9]

**Table 1:** Baseline characteristics of the MST+YOT and YOT samples included in the economic evaluation.

	MST+YOT	YOT
	n = 46	n = 45
Male gender, n (%)	38 (83%)	37 (82%)
Age in years, mean (range)	15 (13 to 17)	15 (13 to 17)
Non-white ethnicity, n (%)	23 (50%)	32 (71%)
Socioeconomic status, mean (sd)*	2.5 (1.7)	2.0 (1.7)
Cost of all offences recorded £, mean (sd)	7,169 (8,302)	6,636 (8,633)
*Violent offences*	6,134 (8,479)	5,452 (8,497)
*Non-violent offences*	1,035 (1,167)	1,184 (1,277)
IPPA score, mean (sd)	96 (22)	98 (21)

### Resource use and costs

Table 2 details the mean number of service contacts, and the associated costs, each participant had with MST therapists and YOT services provided over the 6-month treatment period. On average, 65% of the appointments attended were with social workers, 9% with reparation workers, 7% with drugs workers, 7% with group workers, 6% with parenting workers, 4% with psychologists or other professionals and 2% with connexions workers.

**Table 2:** Service use and corresponding costs (£) by young person during the intervention period.

	MST+YOT		YOT		Mean difference	
	(n = 46)		(n = 45)			
Resource	Number	Cost	Number	Cost	Number	Cost
	Mean (SD)	Mean (SD)	Mean (SD)	Mean (SD)	Mean (95% CI)	Mean (95% CI)
MST	28.6 (18.4)	2285 (1471)	0.0 (0.0)	0 (0)	28.6 (23.1 to 34.0)	2285 (1849 to 2720)
Social worker	10.0 (7.0)	556 (388)	13.9 (10.6)	776 (591)	−3.9 (−7.7 to −0.2)	−220 (−427 to 12)
Reparation worker	1.8 (2.4)	76 (99)	1.5 (2.1)	63 (86)	0.3 (−0.6 to 1.2)	13 (−26 to 51)
Drugs worker	1.0 (1.3)	41 (56)	1.4 (2.8)	59 (115)	−0.4 (−1.3 to 0.5)	−18 (−55 to 20)
Connexions worker	0.6 (1.2)	25 (52)	0.3 (1.1)	14 (46)	0.3 (−0.2 to 0.8)	11 (−9 to 32)
Parenting worker	0.7(2.5)	27 (104)	1.6 (3.3)	68 (138)	−0.9 (−2.2 to 0.2)	−41 (−92 to 10)
Group worker	1.1 (2.1)	13 (26)	1.4 (2.7)	17(33)	−0.3 (−1.3 to 0.7)	−4 (−16 to 9)
Psychologist	0.2 (0.7)	13 (51)	0.3 (0.9)	23 (69)	−0.1 (−0.5 to 0.2)	−10 (−36 to 15)
Other appointments	0.4 (1.1)	15 (45)	0.5 (1.7)	20 (72)	− 0.1 (−0.7 to 0.5)	−5 (−31 to 19)
Total (excluding MST)	15.8 (9.0)	765 (468)	20.9 (13.0)	1040 (671)	−5.4 (−10.0 to 0.7)	−275 (−515 to −34)

Resource use differed little between the two groups. Overall, young persons in the MST+YOT group attended a lower number of appointments, in particular a lower number of appointments with social workers, than those in the YOT group.

The cost of a 60-minute MST session was estimated to be £80 and the total cost of the MST intervention was £2,285 (SD £1,471) per participant with a mean number of sessions attended of 28.6 (SD 18.4).

Total costs per participant over the 18-month follow-up period are reported in Table 3. Results from non-parametric bootstrap replications did not differ substantially from the parametric results and are not reported here. The MST+YOT group were significantly cheaper in terms of YOT services than the YOT group (£3,402 versus £4,619, p = 0.006), but more expensive including the cost of MST, although not significantly so (£5,687 versus £4,619, p = 0.195). The MST+YOT group cost less in terms of the cost of crimes recorded but this difference was not significant (£9,425 versus £11,715, p = 0.456). The mean difference, −£2,290 (95% CI −£9,066 to £4,485) represents the savings in crime generated by the addition of MST to YOT services.

**Table 3:** Cost-offset and sensitivity analyses (£).

	MST+YOT			YOT			
		Cost of services	Cost of crime		Cost of services	Cost of crime	
	n	Mean (SD)	Mean (SD)	n	Mean (SD)	Mean (SD)	Net benefit (95% CI)
18-month follow-up, patients with full data	46	5687 (3045)	9425 (14044)	45	4619 (2979)	11715 (18257)	1222 (−5838 to 8283)
Imputation of missing data using the mean by group	55	5796 (2991)	9032 (12943)	52	4627 (2893)	11461 (17035)	1260 (−4771 to 7291)
Imputing of missing data using the median by group	55	5776 (2993)	8024 (13214)	52	4612 (2894)	10395 (17294)	1207 (−4915 to 7328)
Imputing of missing data using last value carried forward	55	5796 (2993)	8150 (13164)	52	4627 (2894)	10245 (17377)	926 (−5202 to 7054)
30-month follow-up, patients with full data	36	7465 (3577)	12767 (17990)	36	7037 (4271)	15202 (23867)	2007 (−8458 to 12471)

### Cost-offset analysis

**Figure 1:**
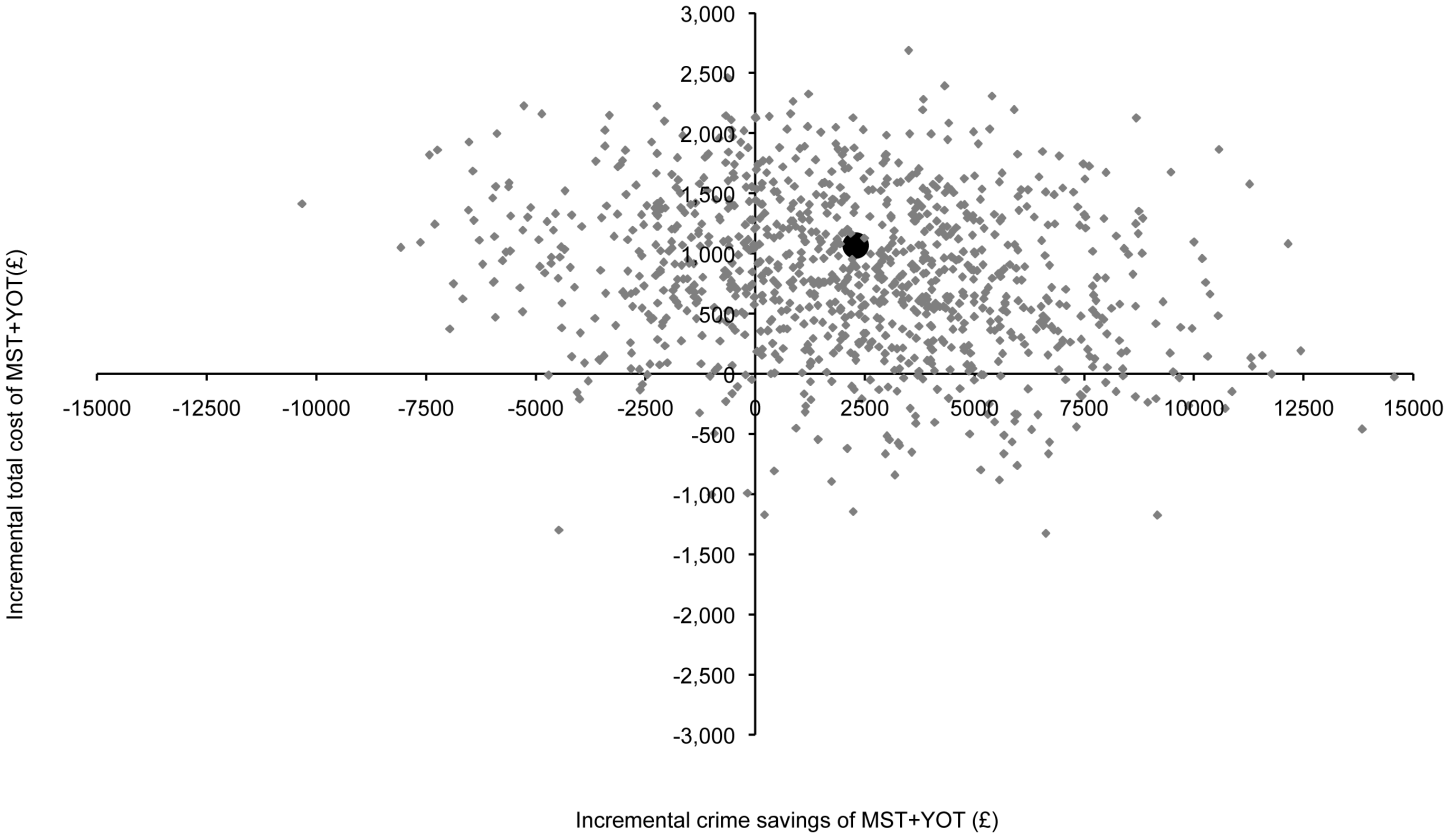
Bootstrapped incremental costs and incremental crime savings of MST+YOT versus YOT.

The net benefit per young person for the 18-month follow-up was estimated to be £1,222 (95% CI −£5,838 to £8,283) (see Table 3). The scatter-plot in Figure 1 shows the bootstrapped replications for incremental service costs and incremental savings in crime and demonstrates that MST+YOT is more costly than YOT alone for almost all replications (points above the x-axis) but is also associated with greater savings related to crime (points to the right of the y-axis). There is a 63% probability that the net-benefit of MST+YOT is positive in favour of the MST+YOT group. This number is calculated by the number of times replications yield a positive net benefit divided by the total number of replications (one thousand). Since this study is a cost-offset analysis, and not a full cost-effectiveness analysis, involving a measure of participant outcome, this scatter plot cannot be viewed as a cost-effectiveness plane, commonly used to support decision making. Instead it is a visual representation of the incremental costs and savings associated with MST+YOT compared to YOT alone.

### Sensitivity analyses

The following sensitivity analyses were undertaken and are reported in Table 3:

- Imputation of missing data using the mean and median by randomisation group and the last value carried forward on the 18-month follow-up data
- Inclusion of participants with full data at the 30-month of follow-up

These analyses did not alter the finding that MST+YOT generates positive net-benefit in comparison to YOT alone.

## Discussion

In this study the cost-offset implications of adding MST to standard YOT services were assessed using data from the first RCT of MST in the UK.[20] The base-case analysis shows that over an 18-month follow-up period, MST+YOT, when compared with YOT alone, has the scope to generate cost-savings through reductions in criminal activity with a probability of 63%. Results were consistent over time, as supported by analysis using the 30-month follow-up data, and were unchanged when missing data were imputed.

This study presents several limitations. First, YOT service use data were only collected in the first year of the study (at base-line and 6-month follow-up). This does not give us the precision needed to conclude about this intervention taking into account the time horizon considered in the economic evaluation (18 months follow-up).

Second, criminal activity is likely to be underestimated as the data only include events that come to the attention of the criminal justice system (court appearances, criminal orders, police custody records, arrests etc). Criminal activity, which remains undetected, but is still associated with costs to society, is excluded. However, this loss has to be balanced against the problems with self-report crime data which are likely to be less accurate than criminal record data.

Thirdly, this study took a narrow economic perspective which included only those services recorded by the YOTs involved in the study. The results therefore exclude contact with any other health, education or social services, which may have been involved in the care of these young people.

Finally, the study was limited by the lack of a generic measure of participant outcome, which restricted the method of economic evaluation to a cost-offset analysis. The ultimate purpose of economic evaluation is to support decisions about the efficient allocation of scarce societal resources between competing objectives.[21], [22] In order to do this, a common unit of outcome is required to allow comparison across diverse interventions. Cost-offset analysis does not allow such comparisons and does not take into account the broader effects of MST on the quality of life of the young people. However, re-offending in this population is still a useful measure because it relates to a key policy objective[23] and captures positive externalities of the intervention.[24]

## Conclusion

Despite the limitations outlined, the findings of the clinical and this economic evaluation provide initial indications that MST may be a promising approach to tackle youth offending in the UK. In the light of the limitations mentioned above, the Department of Health, in conjunction with the Department for Children, Schools and Families, is funding a pragmatic multi-centre RCT (the START trial) to evaluate the effectiveness and cost-effectiveness of MST in a UK context. This study will provide a broader assessment of the effectiveness and cost-effectiveness of MST in a larger population.
